# Therapeutic targeting of STAT3 pathways in pancreatic adenocarcinoma: A systematic review of clinical and preclinical literature

**DOI:** 10.1371/journal.pone.0252397

**Published:** 2021-06-17

**Authors:** Sarah Peisl, Claudia Mellenthin, Lucie Vignot, Carmen Gonelle-Gispert, Leo Bühler, Bernhard Egger

**Affiliations:** 1 Department of Surgery, HFR Fribourg, Fribourg, Switzerland; 2 Department of Oncology, HFR Fribourg, Fribourg, Switzerland; 3 Surgical Research Unit, Faculty of Science and Medicine, University of Fribourg, Fribourg, Switzerland; 4 Faculty of Science and Medicine, University of Fribourg, Fribourg, Switzerland; National University Singapore Yong Loo Lin School of Medicine, SINGAPORE

## Abstract

**Background/Objectives:**

Pancreatic ductal adenocarcinoma is a highly lethal disease with increasing incidence. Due to high resistance, chemo/radiotherapy has limited success in pancreatic cancer and only marginally prolongs patient survival. Therefore, novel biomarkers and therapeutic targets are needed. In the present review, we performed a comprehensive summary of therapeutic approaches targeting the GP130/JAK/STAT3 pathway.

**Methods:**

We systematically reviewed the PubMed and Embase databases for preclinical and clinical studies, from inception to October 4, 2020, on drugs targeting the GP130/JAK/STAT3 pathway. Bias assessments and qualitative analyses were performed.

**Results:**

Twenty-five preclinical and nine clinical trials were included in the review. All preclinical studies reported a favorable outcome in terms of pancreatic ductal adenocarcinoma progression. Futhermore, drugs targeting the GP130/JAK/STAT3 pathway were shown to be efficient chemosensitizers. However, high publication bias was assumed. In the clinical setting, bazedoxifene and itacitinib improved patient outcomes.

**Conclusion:**

Preclinical studies strongly suggest significant efficacy of drugs targeting GP130/JAK/STAT3 in the treatment of pancreatic ductal adenocarcinoma and that these molecules are effective chemosensitizers. Though only a few trials have shown the efficacy in a clinical setting, the STAT3 pathway remains a promising drug target for future treatment of pancreatic ductal adenocarcinoma and may help overcome chemotherapy resistance.

## Introduction

Pancreatic ductal adenocarcinoma (PDAC) is a highly lethal disease with increasing incidence. In most cases, pancreatic cancer presents at an advanced stage, with only 20% of all cases undergoing surgical resection. In terms of prognostic outcomes for patients, pancreatic adenocarcinoma ranks last, with an overall 5-year survival rate of 2–9% [1, 2]. Even though the management of pancreatic adenocarcinoma is evolving with the introduction of novel surgical techniques and medical therapies, only minor improvements in outcomes have been achieved. Due to high resistance, chemotherapy and radiotherapy have limited success in metastatic PDAC and only marginally prolong patient survival [3]. Current treatment options for metastatic PDAC are modified FOLFIRINOX/FOLFIRINOX or nab-paclitaxel and gemcitabine in patients with good performance status, and gemcitabine with or without a second agent for those with poor performance status [4]. Most recently, trials studying the update of immunotherapy in PDAC were negative except in a subgroup of adenocarcinoma with microsatellite instability [5].

Considering the lack of effective treatment, the identification of novel biomarkers and therapeutic targets is fundamental to developing new treatment strategies and improving clinical outcomes. Recent studies suggest that signaling pathways involving STAT3 play a key role in tumorigenesis, progression and drug resistance in several human malignancies such as leukemia, lymphomas as well as solid tumors such as hepatocelullar carcinoma, esophageal, lung, prostate, bladder and breast cancer [6, 7]. Animal models of PDAC have shown that STAT3 is an important regulator of stem cell self-renewal and cancer cell survival [8, 9]. Upregulation of STAT3 has been shown to promote the development of PDAC from pancreatic intraepithelial neoplasia [10, 11], as well as pro-metastatic niche formation in the liver [12]. Furthermore, STAT3 has been shown to mediate resistance to chemotherapy and to be associated with adverse outcomes following resection of PDAC with curative intent [13–15].

**Fig 1:**
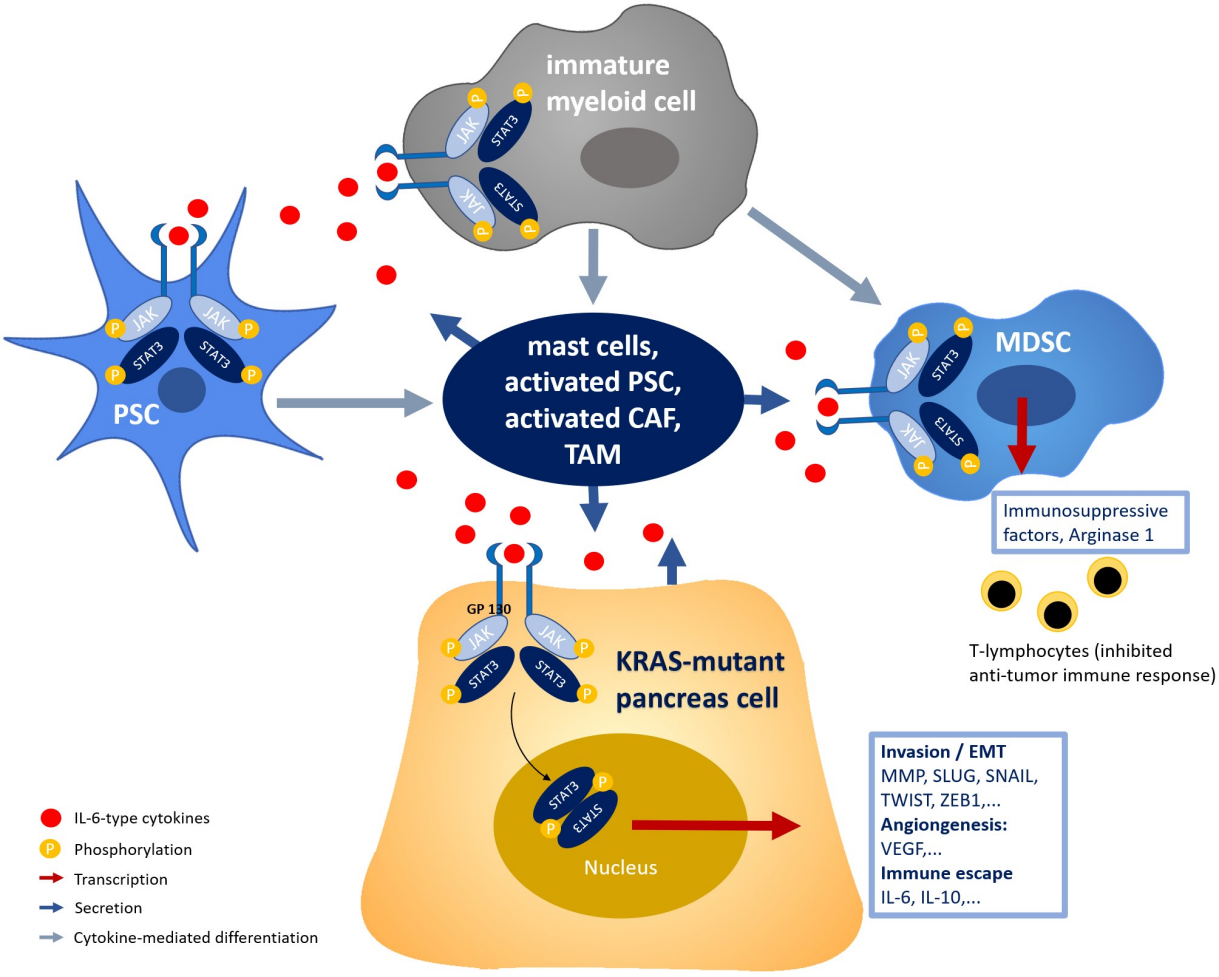
Schematic presentation of IL-6/JAK/STAT3 pathway in pancreatic cancer cells and tumor microenvironment. (PSC: pancreatic stellate cell, CAF: cancer associated fibroblast, TAM: tumor associated macrophages, MDSC: myeloid derived suppressor cells).

As illustrated in Fig 1, IL-6-type cytokines (IL-6, IL-10, IL-11, Leukemia inhibitory factor (LIF), Cardiotrophin-1 (CT-1), Oncostatin-M (OSM), Ciliary neurotrophic factor (CNTF)), bind glycoprotein-130 (GP130) and activate janus kinase (JAK), which in turn phosphorylates STAT3, among other signaling mediators in PDAC tumor cells as well as cells of tumor microenvironment (TME) [16]. TME in PDAC is a complex system which consists, along with extensive stromal networks, of different cell components such as pancreatic stellate cells (PSCs), cancer associated fibroblasts (CAFs), tumor associated macrophages (TAMs), mast cells, regulatory T-cells and myeloid derived suppressor cells (MDSCs), synergizing to support tumor progression, immune evasion and metastatic spreading. Interactions between different cells within the TME are mediated through signaling molecules such as STAT3 activation via IL-6-type cytokines. For instance, PDAC tumor cells can stimulate immune cells to secrete IL-6-type cytokines, supporting the development of immunosuppressive TAMs and MDSCs as well as the activation of PSCs and CAFs, which in turn induce the secretion of inflammatory cytokines through positive feedback loops [11, 17–22]. Thus, STAT3 activation drives immune cells towards immunosuppressive phenotype by inhibiting regulatory T-cells, which in turn sustains tumor immune evasion. Furthermore, the phosphorylation of STAT3 leads to enhanced transcription of downstream target genes, which promote angiogenesis, invasion, and epithelial-mesenchymal transition (EMT) [23].

Accordingly, pathways involving STAT3 appear to be promising drug targets for the treatment of PDAC. In particular, IL-6 has been shown to be a potentially efficient therapeutic approach for overcoming chemotherapy resistance. The purpose of this study was to provide a comprehensive summary of therapeutic approaches targeting the GP130/JAK/STAT3 pathway in pancreatic adenocarcinoma through a systematic qualitative review of the literature.

## Methods

This systematic review was performed in accordance with the Preferred Reporting Items for Systematic Reviews and Meta-Analyses (PRISMA) [24]. Studies were identified by searching PubMed using the following search terms:

(carcinoma, pancreatic ductal[MeSH Terms]) AND (interleukin-6[MeSH Terms])
(carcinoma, pancreatic ductal[MeSH Terms]) AND (jak 2 protein tyrosine kinase[MeSH Terms])
(carcinoma, pancreatic ductal[MeSH Terms]) AND (jak 1 protein tyrosine kinase[MeSH Terms])
(carcinoma, pancreatic ductal[Mesh]) AND (stat3 transcription factor[MeSH Terms])
(carcinoma, pancreatic ductal[MeSH Terms]) AND (gp130, cytokine receptor[MeSH Terms])

Embase was searched using the following search query:

(‘pancreas adenocarcinoma’/exp OR ‘adenocarcinoma, pancreas’ OR ‘pancreatic adenocarcinoma’ OR ‘pancreatic ductal adenocarcinoma’) AND (‘stat3 protein’/exp OR ‘stat3 protein’ OR ‘stat3 transcription factor’ OR ‘protein stat3’ OR ‘signal transducer and activator of transcription 3’ OR ‘stat3’ OR ‘transcription factor stat3’)
(‘pancreas adenocarcinoma’/exp AND ‘interleukin 6’/exp, filter for articles)
(‘pancreas cancer’/exp AND ‘janus kinase 2’/exp, filter for articles)
(‘janus kinase 1’/exp AND ‘pancreas cancer’/exp, filter for articles)
(‘gp130’/exp AND ‘pancreas cancer’/exp, filter for articles)

The electronic search was supplemented by a manual search of the reference lists of relevant articles to identify any studies that may have been missed in the database searches. The original database search was performed on May 5, 2020. The electronic search was updated on October 4, 2020.

Inclusion criteria were defined as all trials studying the pharmacological targeting of the GP130-related cytokine/JAK/STAT3 pathway in pancreatic cancer, including studies on animal models or cell cultures. Only studies with an English abstract were included. Reviews, comments, and conference or meeting abstracts were excluded from the analysis. No restrictions on publication date or publication status were imposed.

After exclusion of duplicates, records identified from the literature search were screened for eligibility independently by the two main authors using the title and abstract in an unblinded manner. Disagreements between the reviewers were resolved by consensus. The full-text of articles meeting the inclusion criteria was assessed by the two main authors and reevaluated for the inclusion criteria. Disagreements were, again, resolved by consensus.

We extracted data using a previously prepared extraction form. The information from each included study on the study design, characteristics of analyzed subjects or trial participants, characteristics of the pharmacological agent studied, type of outcome measures, and outcomes was tabulated. On this basis, we performed a qualitative data synthesis.

We performed a quality assessment of clinical trials according to the ROB tool, which was adapted to match non-randomized clinical trials [25]. For preclinical studies, we used the SYRCLE’s risk of bias tool, which was adapted to match *in vivo* and *in vitro* studies [26]. Results were displayed in an analogous fashion as suggested by Higgins et al for systematic reviews of interventions [25]. Bias assessment was conducted for every study by two independent assessors and disagreements resolved by consensus.

Due to the nature of this study, approval from the local Ethics Committee was not required.

## Results

### Study selection

Our search identified 756 records through the database searches (Embase, Pubmed) and the manual search of the reference lists of relevant articles. Initial screening excluded 689 records, including 145 duplicates. The remaining 67 articles were assessed based on the full text, 29 of which were found to be ineligible due to absence of a tested pharmacological substance or the absence of GP130-related cytokine/JAK/STAT3 pathway targeting.

**Fig 2:**
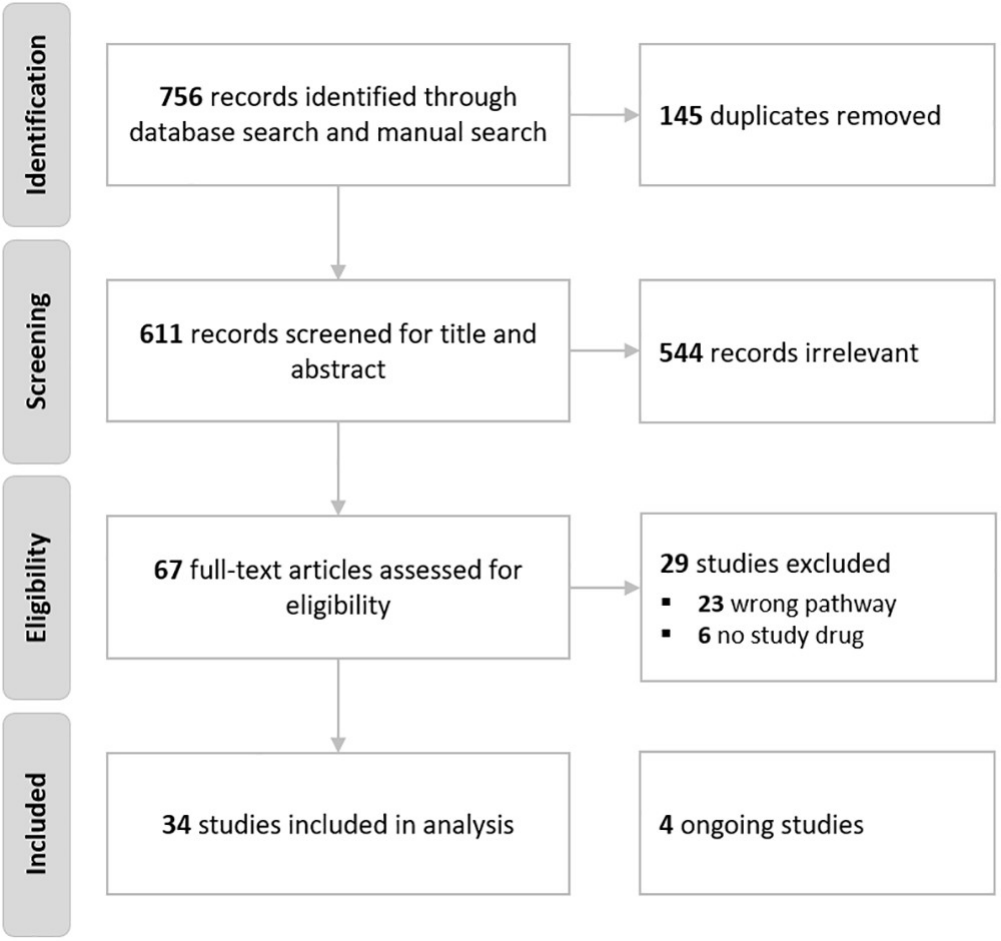
PRISMA flow chart of included studies.

A summary of the study selection process is provided in Fig 2. Ultimately, 38 studies were included in the review, including 4 ongoing trials. All included studies were published in English and no unpublished data were included. No other studies were identified through the electronic search update on October 4, 2020.

### Bias assessment

Table 1 shows the risk of bias assessment for the preclinical studies. Preclinical studies had strong limitations to rigorous bias assessment because few provided sufficient details regarding selection and performance bias. Study protocols were not published beforehand, so a comparison between intended interventions and published interventions was not possible. In animal trials, few studies explicitly stated a randomization process for treatment groups, and treatment results were often assessed manually with semi-quantitative methods. This lack of reporting makes it difficult to accurately determine the risk of bias of the preclinical studies. However, more details were available on the risk of attrition bias, reporting bias, and other bias.

**Table 1:** Bias assessment of preclinical studies.

Reference	Chen 2019	Edderkaoui 2013	Fu 2018	Ge 2015	Goumas 2015	Lin 2010	Long 2017	Palagani 2014	Sahu 2017	Sun 2009	Thoennissen 2009	Wu 2016	Zhang 2018	Nagaraju 2016	Nagaraju 2019	Chen 2016	Lu 2019	Liu 2011	Huang 2016	Luo 2019	Kim 2016	Venkatasubbara 2005	Lu 2017	Liu 2019	Song 2018
	[27]	[28]	[29]	[30]	[31]	[32]	[33]	[34]	[35]	[36]	[37]	[38]	[39]	[40]	[41]	[17]	[42]	[43]	[44]	[45]	[46]	[47]	[48]	[49]	[50]
	T	B	T	T	B	B	V	B	B	T	B	B	T	B	B	T	V	T	B	B	T	T	T	B	B
1) Selection	//	O	//	//	O	O	O	X	O	//	O	X	//	//	O	//	X	//	X	O	//	//	//	O	O
2) Performance	//	X	//	//	X	X	X	X	X	//	X	X	//	X	X	//	//	//	X	X	//	//	//	X	X
3) Detection	X	X	O	O	//	//	//	//	//	O	X	//	O	//	O	//	//	X	X	//	O	O	O	O	//
4) Reporting	O	O	O	O	O	O	O	O	O	O	O	O	O	O	O	O	O	O	O	O	O	O	O	O	O
5) Other	O	O	O	O	O	O	O	O	O	O	O	O	O	O	O	O	O	O	O	O	O	O	O	O	O

1) Was the allocation sequence adequately generated, applied, and concealed? Were the groups similar at baseline or were they adjusted for confounders?

2) Were the caregivers and/or investigators blinded from knowledge of which intervention each animal received during the experiment?

3) Were animals/cell cultures selected at random for outcome assessment? Was the outcome assessor blinded? Was a computed/automatic tool used?

4) Are reports of the study free of selective outcome reporting?

5) Was the study apparently free of other problems that could result in high risk of bias?

V: In vivo study T: in vitro study B: in vivo and in vitro

O Meets criteria (low risk of biais) // Some concerns (unclear risk of bias, insufficient reporting) X Does not meet criteria (high risk of bias)

The quality assessment of the included clinical trials is provided in Table 2. The overall quality of the studies was good, with only one study presenting high risk of selection bias.

**Table 2:** Bias assessment of the clinical studies.

Reference	Burkhardt 2019	Beatty 2019	Ng 2019	Bauer 2018	Hurwitz 2018	Hurwitz 2015	Eckhardt 2009	Macdonald 2005	Cohen 2003
	[51]	[52]	[53]	[54]	[55]	[56]	[57]	[58]	[59]
Design Phase	R	P	P	P	P	P	P	P	P
		Ib/II	I	Ib	III	II	III	II	II
1) Selection process	X	//	//	//	O	O	O	O	O
2) Deviation from intended intervention	O	O	O	O	O	O	O	O	O
3) Missing outcome data	O	O	O	O	O	O	O	O	O
4) Measurement of the outcome	//	O	O	O	O	O	O	O	O
5) Selection of the reported result	O	O	O	O	O	O	O	O	O
6) Overall	//	O	O	O	O	O	O	O	O

1) Does the patient(s) represent(s) the whole experience of the investigator? Is the selection method clear? Was the allocation sequence random?

2) Did the investigator deviate from intended interventions? Were investigators/study participants blinded?

3) Is there evidence that the result was not biased by missing outcome data? Were incomplete outcome data adequately addressed?

4) Was the method of measuring the outcome (in)appropriate? Could measurement or ascertainment of the outcome have differed between intervention groups?

5) Were the data that produced this result analyzed in accordance with a pre-specified analysis plan?

6) Was the study apparently free of other problems that could result in high risk of bias?

R: retrospective P: prospective

O Meets criteria (low risk of bias) // Some concerns (unclear risk of bias, insufficient reporting) X Does not meet criteria (high risk of bias)

### Preclinical studies

As summarized in Table 3, 25 of the included studies were preclinical trials testing 20 substances targeting the GP130/JAK/STAT3-pathway. Twenty-four studies performed *in vitro* experiments using human pancreatic cancer cells [17, 27–32, 34–36, 38–41, 43–47, 49, 50, 60, 61]. *In vivo* experiments were performed in 17 studies using mouse xenograft tumor models (n = 14) [28, 31, 34, 35, 38, 40–42, 44, 45, 48–50, 60], chicken chorio-allantoic membrane xenograft tumor models (n = 1) [32], or KPC mice (n = 2) [33, 45]. All studies reported favorable outcomes in terms of pancreatic cancer cell viability, proliferation, migration, colony formation ability, apoptosis, or effects on downstream target genes, as well as tumor growth, tumor volume, or weight in *in vivo* models. Eight studies analyzed the combinational effect of the investigated drug with chemotherapy (i.e., gemcitabine, paclitaxel, 5-fluorouracil, and oxaliplatin) [31, 33, 38, 40, 41, 45, 49, 60]. In all studies, the inhibitory effect on pancreatic cancer cells by the investigated drug was enhanced by chemotherapy.

**Table 3:** Characteristics of the included preclinical studies.

Reference		Study design	Drug	Mechanism of action	Subject	Number	Outcome
Zhang 2018	[39]	In vitro	AG490 (Tyrphostin B42)	JAK2/STAT3 inhibition	HPCC	-	↓ cell viability, ↓ STAT3 overexpression and phosphorylation, downregulation of target genes
Palagani 2014	[34]	In vitro	AG490	JAK2/STAT3 inhibition	HPCC	-	In vitro: ↓ cell proliferation, ↑ apoptosis
		In vivo	+ GSI IX	+ Notch (Hes1) inhibition	Mouse XTM	20	In vivo: ↓ cell proliferation, ↓ tumor growth
Wu 2016	[38]	In vitro	Bazedoxifene	Inhibitor of IL-6/IL-6R/GP130 complex	HPCC	-	In vitro: ↓ STAT3 phosphorylation, downregulation of target genes, ↓ cell migration
		In vivo	+ Pac		Mouse XTM	8	In vivo: ↓ tumor growth, enhanced effect with Pac
			+ Gem				No significant toxicity
Fu 2018	[29]	In vitro	Bazedoxifene	Inhibitor of IL-6/IL-6R/GP130 complex	HPCC	-	↓ cell viability, ↓ cell migration, ↓ colony formation Enhanced effect with combinational therapy
			+ reparixine				
			+ SCH527123				
Chen 2019	[27]	In vitro	Bazedoxifene	Inhibitor of IL-6/IL-6R/GP130 complex	HPCC	-	↓ cell viability, ↓ cell proliferation, ↓ colony formation
Ge 2015	[30]	In vitro	Cryptotanshinone	STAT3 inhibition	HPCC	-	↑ apoptosis, downregulation of target genes
Thoennissen 2009	[37]	In vitro	Cucurbitacin B	Inhibition of phosphorylation of JAK2 and STAT3	HPCC	-	In vitro: ↓ cell proliferation, ↑ apoptosis, enhanced effect with combinational therapy
		In vivo	+ Gem		Mouse XTM	5	In vivo: ↓ tumor volume, ↓ tumor weight
Sun 2009	[62]	In vitro	Cucurbitacin E	Inhibition of STAT3 phosphorylation	HPCC	-	↓ cell proliferation, ↑ apoptosis
Edderkaoui 2013	[28]	In vitro	Ellagic acid	1) Inhibition of STAT3 phosphorylation	HPCC	-	↓ cell proliferation, ↑ apoptosis by embelin
		In vivo	Embelin	2) inhibition of NF-kB	Mouse XTM	24	Enhanced effect with combinational therapy
Lin 2010	[32]	In vitro	FLLL31	Selective inhibition of JAK2/STAT3(SH2)	HPCC	-	In vitro: ↓ STAT3 phosphorylation, downregulation of target genes, ↑ apoptosis
		In vivo	FLLL 32		Chorio-allantoic membrane XTM	-	In vivo: ↓ tumor volume, ↓ neo-angiogenesis
Nagaraju 2016	[40]	In vitro	Ganetispib	HSP90 und JAK2 inhibition	HPCC	-	In vitro: ↓ cell proliferation
		In vivo	+ Gem/Pac		Mouse XTM	35	In vivo: ↓ tumor growth, enhanced effect with combinational therapy
			+ 5-FU/Ox				
Nagaraju 2019	[41]	In vitro	Ganetispib	HSP90 und JAK2 inhibition	HPCC	-	In vitro: ↓ cell proliferation, ↓ VEGF
		In vivo	+ 5-FU		Mouse XTM	16	In vivo: enhanced effect with combinational therapy, no significant toxicity
Lu 2019	[42]	In vitro	IL-9 antibody	Inhibition of IL-9	HPCC	-	In vitro: ↓ STAT3 phosphorylation, ↓ VEGF
		In vivo			Mouse XTM	48	In vivo: ↓ tumor weight, ↑ survival
Chen 2016	[17]	In vitro	Interleukin 32α	Inhibition of JAK2/STAT3	HPCC	-	Downregulation of target genes
Liu 2011	[43]	In vitro	LLL12	Blocking of IL-6-induced STAT3 phosphorylation	HPCC	-	↓ STAT3 phosphorylation, ↓cell viability
Huang 2016	[44]	In vitro	LTP-1	STAT3 inhibitor	HPCC	-	In vitro: ↓ cell proliferation, ↓ cell viability, ↑ apoptosis
		In vivo			Mouse XTM	40	In vivo: ↓ tumor growth
Kim 2016	[46]	In vitro	Morusin	STAT3 inhibitor	HPCC	-	↓STAT3 phosphorylation, downregulation of target genes, ↑ apoptosis
Luo 2019	[45]	In vitro	Phospho-valproic acid (MDC-1112)		HPCC	-	In vitro: ↓ cell proliferation, ↓ colony formation,
		In vivo	+ Gem				
			+ 5-FU		Mouse XTM	16	↓ invasion, ↑ apoptosis with combinational therapy
			+ abraxane		KPC mice	30	In vivo: ↓ STAT3 phosphorylation, downregulation of target genes, ↓ tumor growth with Gem
Sahu 2017	[35]	In vitro	1) Ponatinib	1) Multi-receptor tyrosine kinase inhibitor	HPCC	-	In vitro: ↓ cell proliferation
		In vivo	2) Cobemetinib	2) MEK inhibitor	Mouse XTM	80	In vivo: -↓ tumor growth, ↑ apoptosis with combinational therapy, safety issues (weight loss)
Lu 2017	[48]	In vitro	Ruxolitinib	JAK1/2 inhibitor	HPCC	-	In vitro: ↓ T cell proliferation
		In vivo			Mouse XTM	30	In vivo: ↓ STAT3 phosphorylation, ↑ cytotoxic T-lymphocyte infiltration and activation
Liu 2019	[49]	In vitro	S-Adenosyl-methionine (SAM)	Inhibition of JAK2/STAT3	HPCC	-	In vitro: ↓ cell proliferation, ↑ apoptosis, ↓ invasion
		In vivo	+ Gem		Mouse XTM	24	In vivo: ↓ tumor weight, ↓ tumor volume, enhanced effect with combinational therapy
Song 2018	[50]	In vitro	SZC015 (oleanolic acid derivative)	Suppression of NFκB and JAK2/STAT3	HPCC	-	In vitro: ↓ cell viability
		In vivo			Mouse XTM	15	In vivo: ↓ JAK2/STAT3 signaling, ↑ apoptosis
Venkatasubbarao 2005	[47]	In vitro	Tipifarnib (R1115777)	Inhibition of STAT3 phosphorylation	HPCC	-	↓ STAT3 phosphorylation
Goumas 2015	[31]	In vitro	Tocilizumab	1) Anti-IL6Rα, humanized monoclonal antibody	HPCC	-	In vitro: ↓ STAT3 phosphorylation
		In vivo	2) sgp130Fc	2) GP130 inhibitor	Mouse XTM	40	In vivo: ↓ tumor growth, ↓ neoangiogenesis, no enhanced effect with Gem, ↓ tumor recurrence and metastasis as adjuvant treatment after surgery
			+ Gem				
			+ surgery				
Long 2017	[33]	In vivo	Tocilizumab	Anti-IL6Rα, humanized monoclonal antibody	KPC mice	-	↓ STAT3 phosphorylation, ↓ cell proliferation,
			+ Gem				↑ apoptosis, enhanced effect with Gem

HPCC: human pancreatic cancer cell, PDAC: pancreatic ductal adenocarcinoma, XTM: xenograft tumor model, Ox: oxaliplatin, Gem: gemcitabine, Pac: paclitaxel, 5-FU: 5-fluorouracil,—indicates no data available

### Clinical studies

As summarized in Table 4, nine of the studies were clinical trials including 880 individuals and assessing 5 drugs. One study performed a retrospective analysis of bazedoxifene, an inhibitor of the IL-6/IL-6R/GP130 complex, in patients with pancreatic (n = 5) or gastric adenocarcinoma (n = 2), showing biological tumor marker reduction in 80% and disease regression on PET-CT in 60% of cases [51]. Icatinib, a selective JAK1 inhibitor, was tested in combination with nab-paclitaxel and gemcitabine, showing a synergistic effect with an overall response rate of 24% with an acceptable safety profile in a phase 1b/2 study [52]. However, this study was terminated early due to negative phase 3 results for JAK1/2 inhibitor ruxolitinib [55]. Momelotinib, a JAK1/2 inhibitor, resulted in a partial response in 28% of patients with previously untreated metastatic PDAC (n = 25) in a phase 1 study. However, no significant difference was reported from treatment with paclitaxel and gemcitabine [53]. Ruxolitinib, a JAK1/2 inhibitor, has been investigated in phase 1b, 2, and 3 clinical trials in combination with capecitabine, gemcitabine, and paclitaxel, revealing no significant difference in overall survival or progression-free survival in patients with PDAC [54–56]. Finally, phase 2 and 3 studies have been performed assessing tipifarnib, an inhibitor of STAT3 phosphorylation that showed no single-agent antitumor activity and no difference in overall survival in combination with gemcitabine [57–59]. Four ongoing clinical trials were found, involving tocilizumab, an anti-IL6Rα antibody with favorable results in preclinical studies [31, 33], and napabucasin, a STAT3 inhibitor that is also under investigation in colorectal cancer [63].

**Table 4:** Characteristics of included clinical studies.

Reference		Study design	Drug	Mechanism of action	Subject	Number	Outcome
Burkhardt 2019	[51]	Retrospective	Bazedoxifene	Inhibitor of IL-6/IL-6R/GP130 complex	PDAC	5	Tumor marker reduction of 80%
					Gastric adenocarcinoma	2	Stability of disease on CT in 60%
							Regression on PET-CT in 60%
Beatty 2019	[52]	Phase 1b/2 dose-finding study	Itacitinib	Selective JAK1-inhibition	Advanced PDAC	46	Terminated early due to futility of JANUS study [55]
			+ paclitaxel		Other advanced solid tumors	9	Acceptable safety profile
			+ gemcitabine				Overall response rate: 24%
Ng 2019	[53]	Phase 1 dose-escalation study	Momelotinib	JAK1/2 inhibitor	Untreated metastatic PDAC	25	No significant increase in PFS or OS
			+ paclitaxel				MTD: not reached
			+ gemcitabine				AE: fatigue (80%), nausea (76%), anemia (68%).
							Partial response in 28%, stable disease in 52%
Hurwitz 2015	[56]	Randomized Phase 2	Ruxolitinib	JAK1/2 inhibitor	Metastatic PDAC after treatment failure with gemcitabine	127	No significant increase in PFS
			+ capecitabine				Significant increase in OS in patients with inflammation compared to placebo (p = 0.011)
							Grade 3 anemia more frequent compared to placebo
Bauer 2018	[54]	Phase 1b dose-finding study	Ruxolitinib	JAK1/2 inhibitor	Untreated advanced PDAC	34	Terminated early due to disease progression in 81%
			+ gemcitabine		Other advanced solid tumors	8	Overall response rate in PDAC: 23.5%
			+ paclitaxel				Acceptable toxicity profile
Hurwitz 2018	[55]	Randomized Phase 3 (JANUS)	Ruxolitinib	JAK1/2 inhibitor	Advanced PDAC	307	Terminated early due to futility
			+ capecitabine				No significant difference in PFS or OS
							Acceptable toxicity profile
Eckhardt 2009	[57]	Randomized Phase 3	Tipifarnib (R115777)	Inhibition of STAT3 phosphorylation	Advanced PDAC	244	No significant difference in survival
			+ gemcitabine				Acceptable toxicity profile
							Most common AE: neutropenia, thrombocytopenia
Macdonald 2005	[58]	Randomized Phase 2	Tipifarnib (R115777)	Inhibition of STAT3 phosphorylation	Untreated advanced PDAC	53	6-month survival rate: 19%
							Median time to treatment failure: 1.4 months
							No single-agent antitumor activity
Cohen 2003	[59]	Randomized Phase 2	Tipifarnib (R115777)	Inhibition of STAT3 phosphorylation	Untreated advanced PDAC	20	100% progression at 6 months
							6-month survival rate: 25%
							No single-agent antitumor activity

PFS: progression-free survival, OS: overall survival, MTD: maximum tolerable dose, AE: adverse event, PDAC: pancreatic ductal adenocarcinoma,—indicates no data available

## Discussion

The present systematic review of 25 preclinical studies and 9 clinical trials revealed a good overall effect of the investigated drugs targeting the GP130/JAK/STAT3 pathway in the treatment of PDAC. Table 5 summarizes the outcome and the state of research for each assessed drug. Favorable outcomes have been reported for all 20 drugs investigated in a preclinical setting. Even though these substances appear promising in the treatment of PDAC, only five of these drugs have been investigated in clinical trials. Favorable outcomes and acceptable toxicity profiles have been found in studies investigating bazedoxifene and itacitinib [51, 52]. Notably, bazedoxifene is already approved for the treatment of osteoporosis [64], and itacitinib has been shown to have great potential in recent clinical trials studying the treatment of connective tissue diseases and graft-versus-host disease, among others [65–67].

**Table 5:** Summary of findings by drug.

Drug	Mechanism of action	Outcome	State of research
Bazedoxifene	Inhibitor of IL-6/IL-6R/GP130 complex	Positive	Clinical study, retrospective
		Synergism with paclitaxel and gemcitabine	
Ganetespib	HSP90/JAK2	Positive	Preclinical research
		Synergism with gemcitabine/paclitaxel and 5-fluorouracil/oxaliplatin	
Ruxolitinib	JAK1/2 inhibitor	Negative in combination with gemcitabine/paclitaxel	Phase 1b clinical trial
		Negative in combination with capecitabine	Phase 2+3 clinical trial
Tipifarnib (R1115777)	Inhibition of STAT3 phosphorylation	Negative as single agent	Phase 2 clinical trials
		Negative in combination with gemcitabine	Phase 3 clinical trial
Momelotinib	JAK1/2 inhibitor	Negative	Phase 1 clinical trial
		Negative in combination with gemcitabine and paclitaxel	
Itacitinib	Selective JAK-1 inhibition	Positive	Phase 2 clinical trial
AG490	JAK2 inhibitor	Positive	Preclinical research
Cryptotanshinone	STAT3 inhibition	Positive	Preclinical research
Cucurbitacin B	Inhibition of phosphorylation of JAK2 and STAT3	Positive; synergism with gemcitabine	Preclinical research
Cucurbitacin E	Inhibition of STAT3 phosphorylation	Positive	Preclinical research
Ellagic acid	Inhibition of STAT3 phosphorylation	Positive	Preclinical research
FLLL31/32	Selective JAK2/STAT3(SH2) inhibition	Positive	Preclinical research
IL-32α	Inhibition of JAK2/STAT3	Positive	Preclinical research
IL-9 antibody	IL-9 inhibition	Positive	Preclinical research
LLL12	Blocking of IL-6-induced STAT3 phosphorylation	Positive	Preclinical research
LTP-1	STAT3 inhibitor	Positive	Preclinical research
Morusin	STAT3 inhibitor	Positive	Preclinical research
Phospho-valproic acid (MDC-1112)	STAT3 inhibitor	Positive; synergism with gemcitabine	Preclinical research
Ponatinib	Multi-receptor tyrosine kinase inhibitor	Positive	Preclinical research
S-Adenosylmethionine (SAM)	Inhibition of JAK2/STAT3	Positive; synergism with gemcitabine	Preclinical research
SZC015	Suppression of NFκB and JAK2/STAT3	Positive	Preclinical research
Tocilizumab	Anti-IL6Rα, humanized monoclonal antibody	Positive; synergism with gemcitabine	Preclinical research
			Ongoing clinical trials (NCT04258150, NCT02767557)
Napabucasin	STAT3 inhibitor	Ongoing	Ongoing phase 1 and 3 clinical trials (NCT02231723, NCT02993731)

Even though the PDAC tumor micro-environment (TME) has been shown to be a promising target for improving PDAC treatment, none of the included studies in this systematic review examined the influence of the analyzed substances on stromal or immune cells.

The TME plays an important role in tumorigenesis and chemoresistance by close interaction with tumor cells. Furthermore, TME has been shown to be highly immunosuppressive, promoting immune evasion, hence sustaining tumor progression [18–20]. Immunotherapy, so far, has not demonstrated substantial clinical improvement as single agent in the treatment of PDAC [5]. Therefore, strategies simultaneously targeting PDAC tumor cells as well as different immune checkpoints might be needed. The interactions of PDAC tumor cells and different cells within the TME such as CAFs, MDSCs, TAMs, are mediated through GP130/JAK/STAT3 pathway [11, 17–20, 68]. STAT3 inhibition might thus have consequences in shaping TME towards anti-tumor phenotype by acting on both immune and tumor cells [18–20]. In combination with chemotherapeutic agents and immunotherapy, it might significantly increase therapeutic efficacy in the treatment of PDAC.

Recent studies have shown the important role of the STAT3 pathway in tumorigensis, as well as the STAT3-mediated resistance to chemotherapy in *in vivo* models of PDAC [8–15]. The results from the preclinical trials presented in this review confirmed the importance of the GP130/JAK/STAT3 pathway in PDAC and its role as a possible drug target. Furthermore, several of the studies showed a synergy between the investigational drug and chemotherapy, such as gemcitabine, paclitaxel, 5-fluorouracil, and oxaliplatin [31, 33, 38, 40, 41, 45, 49, 53–55, 57, 60]. However, to the best of our knowledge, drugs targeting GP130/JAK/STAT3 have never been studied as chemosensitizers in addition to the currently emerging FOLFIRINOX regimen [4]. Even though some promising outcomes have been shown in clinical trials [51, 52], several studies were terminated prematurely due to high progression rates and futility. This may reflect the difficulty showing a significant benefit in patients presenting with PDAC, as it is known to be a highly lethal disease that is often diagnosed at an advanced stage and has a poor prognosis with an overall 5-year survival rate of 2–9% [1, 2].

The discrepancy between preclinical and clinical data may also result from the fact that, in contrast to the preclinical studies, the clinical trials did not verify the activation of the STAT3 pathway in PDAC. The benefit of targeted GP130/JAK/STAT3 therapy may be increased by selecting patients with previously known STAT3 pathway activation in PDAC cells.

The present systematic review included all preclinical and clinical trials of drugs targeting the GP130/JAK/STAT3 pathway. Furthermore, we searched for ongoing, unpublished trials, leading to a thorough analysis of the current state of research. However, because all published preclinical studies reported a positive outcome, we suspect that several negative studies may not have been published and concluded relevant publication bias, leading to an overestimation of the effect of GP130/JAK/STAT3-targeting drugs in the treatment of PDAC in the preclinical setting. Furthermore, the substantial heterogeneity among the preclinical and clinical studies did not allow a quantitative analysis or measurement of the effect size.

## Conclusion

Preclinical studies strongly suggest significant efficacy of drugs targeting GP130/JAK/STAT3 in the treatment of PDAC and that these molecules are effective chemosensitizers, possibly through simultaneous effect on tumor cells and TME. Though only a few trials have shown the efficacy in a clinical setting, the GP130/JAK/STAT3 pathway remains a promising drug target for the development of future treatments for PDAC and may help overcome chemotherapy resistance.

## Supporting information

S1 Checklist (DOC)
